# Prognostic implications of quantified coronary atherosclerosis and myocardial perfusion in diabetes

**DOI:** 10.1186/s12933-025-03006-x

**Published:** 2025-12-02

**Authors:** Matias Mäenpää, Ruurt A. Jukema, Pepijn van Diemen, Sarah Bär, Pieter G. Raijmakers, Ralf Sprengers, Roel S. Driessen, Jeroen J. Bax, Paul Knaapen, Juhani Knuuti, Ibrahim Danad, Antti Saraste, Teemu Maaniitty

**Affiliations:** 1 Turku PET Centre, Turku University Hospital and University of Turku, P.O. Box 52, 20521 Turku, Finland; 2 Department of Cardiology, Amsterdam UMC, Vrije Universiteit Amsterdam, Amsterdam, The Netherlands; 3 Department of Cardiology, Bern University Hospital Inselspital, Bern, Switzerland; 4 Department of Cardiology, Leiden University Medical Center, Leiden, The Netherlands; 5 Department of Clinical Physiology, Nuclear Medicine, and PET, Turku University Hospital, Turku, Finland; 6 Department of Cardiology, Radboud University Medical Center, Nijmegen, The Netherlands; 7 Heart Center, Turku University Hospital and University of Turku, Turku, Finland

**Keywords:** Cardiac complications, Coronary artery disease, Coronary computed tomography angiography, Diabetes, Positron emission tomography, Prognosis

## Abstract

**Background:**

Coronary artery disease (CAD) is a major contributor to cardiovascular events in individuals with diabetes. Quantification of coronary atherosclerotic burden is now feasible from coronary computed tomography angiography (CTA) whereas positron emission tomography (PET) enables quantitative assessment of myocardial perfusion. We studied the prognostic implications of quantitatively measured coronary plaque burden and myocardial perfusion in diabetic vs. non-diabetic patients with suspected CAD.

**Methods:**

In this observational cohort study, 1311 symptomatic patients with suspected CAD underwent coronary CTA and [^15^O]H_2_O PET perfusion imaging. Coronary plaque burden was quantified using artificial intelligence–based analysis and reported as percent atheroma volume (PAV). Myocardial perfusion was assessed as regional stress myocardial blood flow (sMBF), with abnormal perfusion defined as ≥ 2 adjacent segments with sMBF < 2.3 ml/g/min. The composite endpoint was all-cause death, myocardial infarction (MI), or unstable angina pectoris (UAP) over 7 years.

**Results:**

Among the 1311 patients, 251 (19%) had diabetes and 134 (10%) experienced an adverse event during follow-up. The annual event rate was low (0.8% [95% CI 0.6–1.1%]) in non-diabetic patients with normal myocardial perfusion and increased significantly with the presence of either diabetes (2.3% [95% CI 1.4–3.8%]), abnormal perfusion (2.6% [95% CI 2.1–3.3%]), or both (3.2% [95% CI 2.1–4.8%]) (*p* < 0.001). Among patients with normal myocardial perfusion, those with diabetes had two-fold PAV as compared with non-diabetic individuals (median 8.2% vs. 4.1%, *p* < 0.001). In multivariable Cox regression models, both PAV (HR 1.03 [95% CI 1.01–1.05] per 1% increase, *p* < 0.001) and regional sMBF (HR 1.04 [95% CI 1.01–1.07] per 0.1 ml/g/min decrease, *p* = 0.016) were independent predictors of adverse outcome in non-diabetic patients. In diabetic patients, only PAV (HR 1.04 [95% CI 1.01–1.07], *p* = 0.014) was predictive, whereas sMBF was not.

**Conclusions:**

Coronary atherosclerotic plaque burden appears as an important predictor of long-term cardiovascular outcomes both in diabetic and non-diabetic patients. In patients with diabetes, normal myocardial perfusion does not necessarily imply low event risk, partly attributable to higher coronary plaque burden. Quantitative imaging methods for detailed CAD phenotyping shed light on the complex relationship between diabetes and clinical outcomes.

**Graphical abstract:**

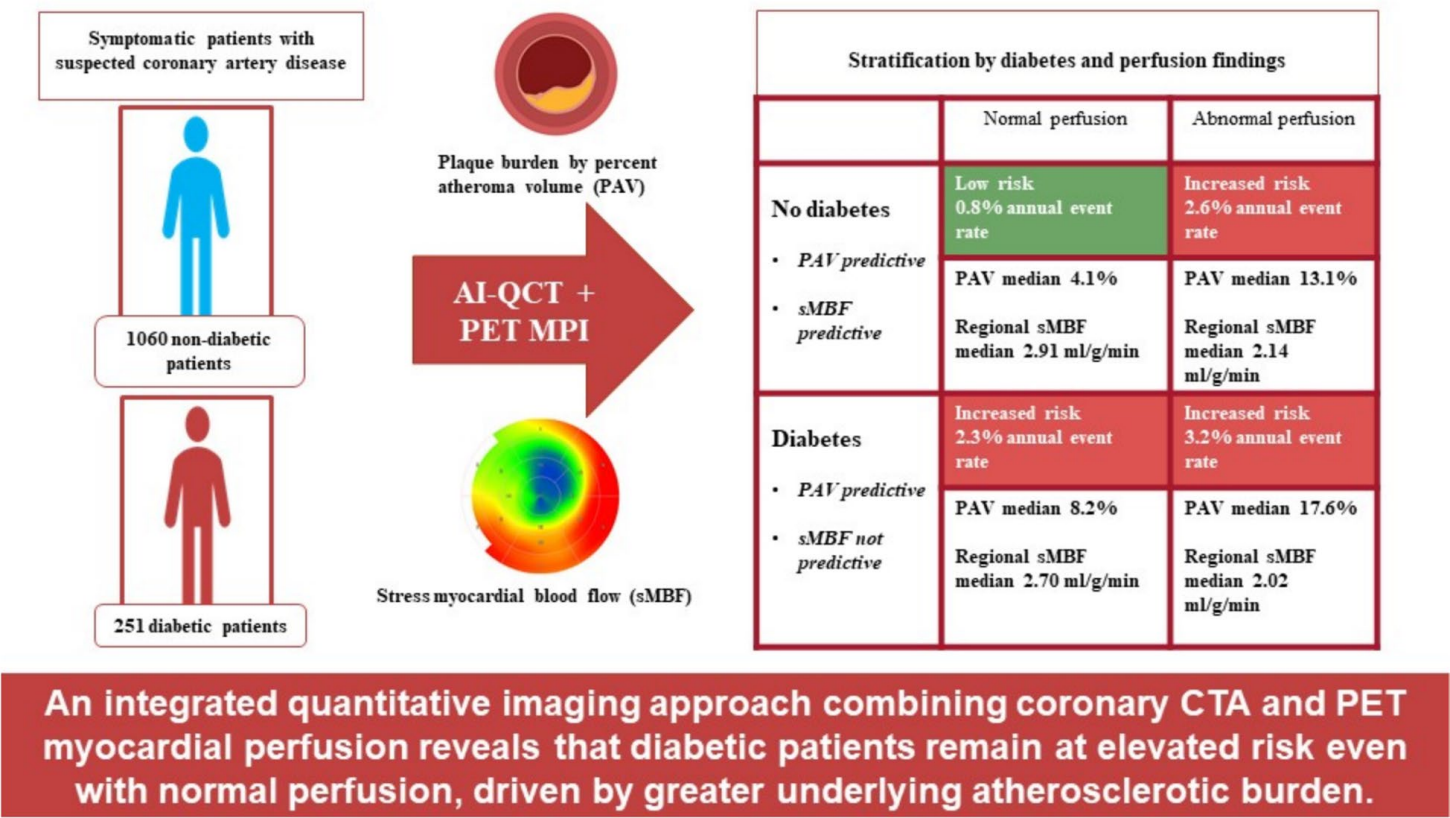

**Supplementary Information:**

The online version contains supplementary material available at 10.1186/s12933-025-03006-x.

## Research insights

**What is currently known about this topic?**

- Diabetes raises cardiovascular risk. CTA and PET assess coronary disease from different perspectives. Prognostic roles of plaque burden vs perfusion are unclear in diabetes.

**What is the key research question?**

- Does plaque burden and perfusion imaging predict cardiovascular risk differently in diabetic vs non-diabetic patients?

**What is new?**

- In non-diabetics, both plaque burden and perfusion predict risk. In patients with diabetes, normal myocardial perfusion does not necessarily imply low event risk, partly attributable to higher coronary plaque burden.

**How might this study influence clinical practice?**

- These findings emphasize the importance of coronary plaque burden both in diabetic and non-diabetic patients. Normal myocardial perfusion in diabetic patients does not necessarily imply low event risk.

## Background

Patients with diabetes mellitus are at increased risk for developing coronary artery disease (CAD) and cardiovascular events [1, 2]. Furthermore, the incidence of diabetes has surged significantly over past decades and myocardial infarction (MI) is the most common cause of death among diabetic patients [3, 4]. Previously, patients with diabetes but without prior MI were reported to have similar risk of cardiovascular mortality as patients with a history of MI [5]. More recently, this hypothesis has been questioned [6], and large-scale real-world data in the era of effective preventive therapy suggest that patients with diabetes might have similar risk of cardiovascular events as patients without diabetes in the absence of obstructive CAD [7, 8]. However, conflicting evidence also exists, highlighting the potential prognostic significance of non-obstructive CAD in diabetes [9]. Not surprisingly, tools for prognostic assessment of CAD in patients with diabetes remain of great interest [10].

Myocardial perfusion imaging (MPI) is a well-established non-invasive functional imaging modality for diagnosis, risk stratification, and guiding treatment of CAD [11, 12]. Quantitative MPI by positron emission tomography (PET) allows measurement of global and regional myocardial blood flow (MBF), integrating the hemodynamic effects of epicardial CAD and coronary microvascular dysfunction, with proven prognostic value [13–17]. Over the last decade, coronary computed tomography angiography (CTA) has become a routine clinical non-invasive imaging tool and is now often a first-line diagnostic test for patients with suspected chronic CAD [11, 18]. Recent technological advancement has enabled accurate quantitative assessment of CAD from CTA, including coronary artery stenosis severity and atherosclerotic plaque burden, with recently demonstrated prognostic value [19, 20]. Anatomical and functional imaging modalities, such as coronary CTA and PET MPI can provide complementary diagnostic and prognostic information to guide management of CAD [21]. However, the relative prognostic value of quantified coronary atherosclerosis burden and abnormal myocardial blood flow in non-diabetic vs. diabetic patients remains unknown.

Therefore, we studied the long-term clinical outcome of diabetic vs. non-diabetic patients undergoing detailed imaging phenotyping of CAD, including quantification of non-obstructive and obstructive CAD by CTA and quantitative PET MPI, with an aim to increase understanding about the mechanisms underlying the higher cardiovascular risk in patients with diabetes.

## Methods

### Patients

A total of 1547 consecutive symptomatic patients were identified who had undergone both coronary CTA and PET MPI due to suspected chronic CAD (low to intermediate pre-test probability) at the Turku University Hospital, Finland (n = 875) or Amsterdam University Medical Center, the Netherlands (n = 672) from 2007 to 2016. At Turku University Hospital, patients selectively underwent PET MPI in case that coronary CTA showed suspected obstructive (≥ 50%) coronary stenosis diameter in visual analysis, whereas in Amsterdam University Medical Center patients underwent both coronary CTA and PET MPI irrespective of the imaging findings [22, 23].

**Fig. 1:**
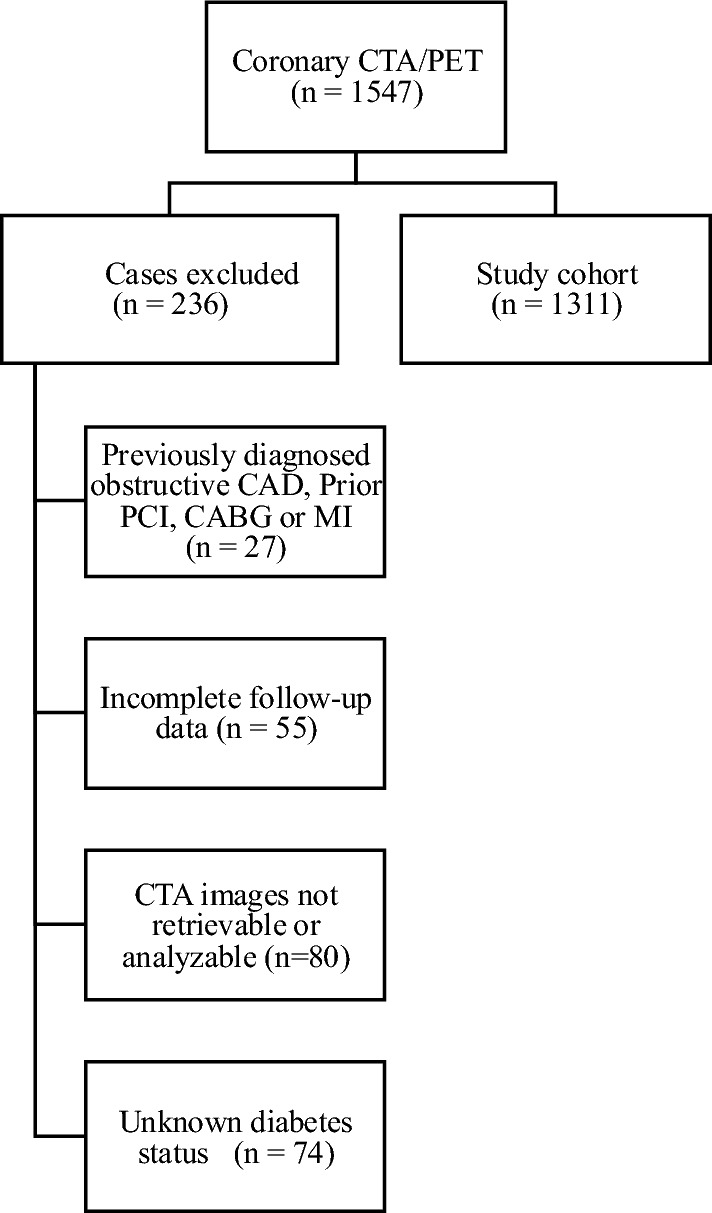
Patient inclusion/exclusion criteria. CTA computed tomography angiography, PET positron emission tomography, CAD coronary artery disease, PCI percutaneous coronary intervention, CABG coronary artery bypass graft, MI myocardial infarction

Patients with prior known obstructive CAD, MI, percutaneous coronary intervention (PCI), or coronary artery bypass grafting (CABG) were excluded (n = 27) as well as patients lost to follow-up (n = 55) or coronary CTA images not retrievable/analyzable (n = 80). Additionally, we excluded 74 patients in whom the presence or absence of diabetes could not be verified. Diabetes was defined as a prior diagnosis of any type of diabetes mellitus (type 1, type 2, or other) based on electronic medical records, the use of glucose-lowering therapy, plasma fasting glucose ≥ 7.0 mmol/l, 2-h plasma glucose ≥ 11.1 mmol/l, or hemoglobin A1c ≥ 6.5%/ 48 mmol/mol [24]. Consequently, the final study cohort consisted of 1311 patients with known diabetes status, fully characterized CAD phenotype by CTA and PET imaging, and complete follow-up data. (Fig. 1**).**

### Image acquisition

The coronary CTA and PET imaging procedures have been previously described in detail [25, 26]. Coronary CTA and PET perfusion scans were performed with a 64-row hybrid PET-CT scanner (GE Discovery VCT or GE D690, General Electric Medical Systems, Waukesha, Wisconsin; Gemini TF 64, Philips Healthcare, Best, the Netherlands) or a 256-row CT scanner (Philips Brilliance iCT, Philips Healthcare, Best, the Netherlands). Sublingual/oral nitrate was administered prior to coronary CTA, and intravenous metoprolol (up to 30 mg) was administered if needed to achieve target heart rate of < 60 beats/min. Coronary CTA was performed using intravenously administered low-osmolar iodine contrast agent. Prospectively triggered acquisition was applied whenever feasible. The CTA acquisition parameter details have been previously reported [27]. Dynamic [^15^O]H_2_O PET myocardial perfusion scan was performed during adenosine vasodilator stress (140 µg/kg/min) [25, 26].

### Image analysis and interpretation

Coronary CTA scans were analyzed using an artificial intelligence-guided quantitative computed tomography (AI-QCT) algorithm (Cleerly Labs, Cleerly, Inc., Denver, CO, USA), that is a U.S. Food and Drug Administration-cleared software validated against expert readers and invasive quantitative coronary angiography (ICA) [19, 28–30]. This software enables automated analysis of coronary CTA using validated convolutional neural networks as previously described comprehensively [19]. A diameter stenosis of ≥ 50% was considered obstructive. Patient-level total coronary atherosclerotic plaque volume was normalized to vessel volume, reported as percent atheroma volume (PAV) to diminish the effect of body size [31]. Additionally, total plaque was divided into non-calcified (HU ≤ 350) and calcified (HU > 350) components and similarly normalized to vessel volume: percent non-calcified plaque volume (NCPV) and percent calcified plaque volume (CPV) respectively. Low-density plaque volume was extremely low and was calculated into non-calcified plaque. In-house developed software (CardiacVUer, Amsterdam UMC, Vrije Universiteit Amsterdam, The Netherlands and Carimas software, Turku PET Centre, Turku, Finland) allowed for the generation of parametric images of quantitative hyperaemic MBF in mL/min/g for each of the 17 left ventricle segments according to the standard American Heart Association model [32]. Presence of abnormal myocardial PET perfusion was defined as the presence on ≥ 2 adjacent segments with stress MBF < 2.3 ml/g/min, indicative of myocardial ischemia [33]. Global stress MBF was reported for the whole left ventricular myocardium and regional stress MBF was reported as the lowest average of two adjacent segments (perfusion defect flow).

### Clinical and follow-up data

Clinical data on diagnosis of diabetes, cardiovascular risk factors, symptoms, and medication were collected from electronic medical records. Early myocardial revascularization was defined as either PCI or CABG within 6 months following CTA/PET imaging, and these were predominantly planned based on CTA/PET imaging findings. Long-term follow-up data were gathered using electronic medical records, standardized telephonic follow-up, and national registry databases, and the events were manually confirmed by the investigators. The primary endpoint was the composite of all-cause death, MI, and unstable angina pectoris (UAP). MI and UAP were defined according to the ESC guideline criteria based on clinical presentation, ECG findings, and biomarker evidence. [34].

### Statistical analysis

Continuous variables were non-normally distributed and are shown as median (25–75th percentile). Categorical variables are shown as counts and percentages. Continuous variables were compared using ANOVA (post-hoc Tukey) or Kruskal–Wallis test (post-hoc Mann–Whitney U test with Bonferroni correction), whereas categorical variables were compared with chi square test (post-hoc Bonferroni). Additionally, continuous variables were analyzed using two-way ANOVA to assess the main and interaction effects of diabetes and perfusion status. Survival curves were created based on Kaplan–Meier estimates and were compared with Mantel-Cox pooled and pairwise log-rank test. Cox proportional hazards model was applied to identify the predictors of composite adverse endpoint of mortality, MI or UAP, including tests for statistical interaction. The follow-up was truncated at 7.0 years due to limited number of remaining patients. Considering a limited number of events, multivariable Cox regression models were constructed using a parsimonious approach based on clinical reasoning and significant univariable associations. However, only one CTA-based variable (i.e., PAV) and one PET-based variable (i.e., regional stress MBF) at a time were included in the multivariable models to avoid multicollinearity. The proportional-hazards assumption was assessed using time-dependent covariate interactions with log(time). A landmark analysis restricted to 0.5–7 years of follow-up was performed, excluding patients who experienced adverse event in the early follow-up (i.e., within 6 months after CTA/PET). Annual event rates for the composite endpoint were calculated and compared by using Poisson regression (post-hoc Bonferroni). Two-sided *p*-value < 0.05 was considered statistically significant. The statistical analyses were conducted with IBM SPSS Statistics version 29.

## Results

Patient characteristics according to diabetes and myocardial perfusion findings are described in Table 1 and Supplementary Table 1. Among the 1311 patients, 584 (45%) had abnormal myocardial perfusion and 251 (19%) had diabetes. More specifically, 605 (46%) patients had normal perfusion and no diabetes, 122 (9%) patients had normal perfusion and diabetes, 455 (35%) had abnormal perfusion and no diabetes, and 129 (10%) had abnormal perfusion and diabetes.

**Table 1:** Patient characteristics according to perfusion findings and diabetes

Model	Total cohort	No diabetes and normal perfusion (0)​	Diabetes and normal perfusion (1)​	No diabetes and abnormal perfusion (2)​	Diabetes and abnormal perfusion (3)​	Overall *p*-value	Pairwise *p*-value (0vs1)	Pairwise *p*-value (0vs2)	Pairwise *p*-value (0vs3)	Pairwise *p*-value (1vs2)	Pairwise *p*-value (1vs3)	Pairwise *p*-value (2vs3)
Patient characteristics												
N	1311 (100%)	605 (46%)	122 (9%)	455 (35%)	129 (10%)							
Abnormal perfusion	584 (45%)											
Diabetes	251 (19%)											
Baseline characteristics												
Age (years)	63 (56–69)	62 (55–68)	63 (58–69)	62 (56–69)	65 (58–69)	0.237						
						Two-way ANOVA	Diabetes *p* = 0.047		Abnormal perfusion *p* = 0.661		Diabetes*Abnormal perfusion *p* = 0.755	
BMI (kg/m^2^)	27.1 (24.6–30.1)	26.2 (23.8–29.1)	29.7 (26.7–34.7)	26.6 (24.6–29.6)	29.8 (27.7–33.7)	< 0.001	< 0.001	0.79	< 0.001	< 0.001	1	< 0.001
						Two-way ANOVA	Diabetes *p* < 0.001		Abnormal perfusion *p* = 0.311		Diabetes*Abnormal perfusion *p* = 0.655	
Male sex	713 (54.4%)	236 (39.0%)	61 (50.0%)	323 (71.0%)	93 (72.1%)	< 0.001	0.144	< 0.001	< 0.001	< 0.001	< 0.001	1
Hypertension	767 (58.5%)	325 (53.7%)	94 (77.0%)	251 (55.2%)	97 (75.2%)	< 0.001	< 0.001	1	< 0.001	< 0.001	1	< 0.001
Dyslipidemia	757 (57.7%)	314 (51.9%)	85 (69.7%)	264 (58.0%)	93 (72.1%)	< 0.001	< 0.001	0.288	< 0.001	0.114	1	0.024
Family history	664 (50.6%)	340 (56.2%)	48 (39.3%)	229 (50.3%)	47 (36.4%)	< 0.001	< 0.001	0.348	< 0.001	1	1	0.03
Current smoker	285 (21.7%)	121 (20.0%)	23 (18.9%)	112 (24.6%)	29 (22.5%)	0.269						
Typical angina pectoris	392 (29.9%)	155 (25.6%)	30 (24.6%)	166 (36.5%)	41 (31.8%)	< 0.001	1	< 0.001	0.906	0.084	1	1
Baseline medications												
Beta-blocker	745 (56.8%)	313 (51.7%)	77 (63.1%)	272 (59.8%)	83 (64.3%)	0.005	0.126	0.054	0.054	1	1	1
Lipid-lowering drug	755 (57.6%)	297 (49.1%)	89 (73.0%)	269 (59.1%)	100 (77.5%)	< 0.001	< 0.001	0.006	< 0.001	0.03	1	< 0.001
Antiplatelet drug	817 (62.3%)	351 (58.0%)	73 (59.8%)	305 (67.0%)	88 (68.2%)	0.01	1	0.018	0.192	0.828	0.996	1
Long-acting nitrate	81 (6.2%)	28 (4.6%)	16 (13.1%)	24 (5.3%)	13 (10.1%)	< 0.001	< 0.001	1	0.084	0.012	1	0.288
ACEi or ARB	538 (41.0%)	197 (32.6%)	76 (62.3%)	181 (39.8%)	84 (65.1%)	< 0.001	< 0.001	0.09	< 0.001	< 0.001	1	< 0.001
Calcium channel blocker	301 (23.0%)	111 (18.3%)	41 (33.6%)	108 (23.7%)	41 (31.8%)	< 0.001	< 0.001	0.192	< 0.001	0.162	1	0.384
Imaging findings												
Regional sMBF (ml/g/min)	2.32 (1.58–2.95)	2.91 (2.54–3.41)​	2.70 (2.43–3.16)​	1.51 (1.07–1.87)​	1.49 (1.03–1.85)​	< 0.001​	0.614​	< 0.001​	< 0.001​	< 0.001​	< 0.001​	1​
						Two-way ANOVA	Diabetes *p* = 0.041		Abnormal perfusion *p* < 0.001		Diabetes*Abnormal perfusion *p* = 0.051	
Global sMBF (ml/g/min)	3.00 (2.22–3.75)	3.67 (3.22–4.28)​	3.55 (3.08–4.12)​	2.14 (1.72–2.57)​	2.02 (1.65–2.42)​	< 0.001​	1	< 0.001​	< 0.001​	< 0.001​	< 0.001​	1​
						Two-way ANOVA	Diabetes *p* = 0.015		Abnormal perfusion *p* < 0.001		Diabetes*Abnormal perfusion *p* = 0.429	
Stenosis degree diameter (%)	42 (18–64)	26 (11–47)​	36 (17–55)​	61 (38–76)​	62 (41–75)​	< 0.001​	0.017​	< 0.001​	< 0.001​	< 0.001​	< 0.001​	1​
						Two-way ANOVA	Diabetes *p* = 0.017		Abnormal perfusion *p* < 0.001		Diabetes*Abnormal perfusion *p* < 0.041	
Obstructive (≥ 50%) stenosis	590 (45.0%)	147 (24.3%)	45 (36.9%)	305 (67.0%)	93 (72.1%)	< 0.001​	0.024	< 0.001	< 0.001	< 0.001	< 0.001	1
PAV (%)	7.8 (2.6–16.5)	4.1 (1.4–9.0)​	8.2 (3.3–15.5)​	13.1 (6.7–22.7)​	17.6 (8.8–28.0)​	< 0.001​	< 0.001​	< 0.001​	< 0.001​	0.001​	< 0.001​	0.121​
						Two-way ANOVA	Diabetes *p* < 0.001		Abnormal perfusion *p* < 0.001		Diabetes*Abnormal perfusion *p* = 0.724	
NCPV (%)	5.1 (2.1–9.1)	2.8 (1.2–5.4)	5.4 (2.5–8.7)	7.9 (4.6–12.1)	9.2 (5.9–15.0)	< 0.001	< 0.001	< 0.001	< 0.001	< 0.001	< 0.001	0.076
						Two-way ANOVA	Diabetes *p* < 0.001		Abnormal perfusion *p* < 0.001		Diabetes*Abnormal perfusion *p* = 0.494	
CPV (%)	2.0 (0.2–6.2)	0.8 (0.0–2.9)	2.8 (0.5–6.4)	3.9 (0.8–9.4)	5.5 (1.4–11.9)	< 0.001	< 0.001	< 0.001	< 0.001	0.178	0.003	0.198
						Two-way ANOVA	Diabetes *p* < 0.001		Abnormal perfusion *p* < 0.001		Diabetes*Abnormal perfusion *p* = 0.968	
Follow-up (up to 7 years)												
Early PCI OR CABG*	269 (20.5)	20 (3.3%)	9 (7.4%)	183 (40.2%)	57 (44.2%)	< 0.001	0.216	< 0.001	< 0.001	< 0.001	< 0.001	1
Early PCI*	201 (15.3%)	20 (3.3%)	9 (7.4%)	134 (29.5%)	38 (29.5%)	< 0.001	0.216	< 0.001	< 0.001	< 0.001	< 0.001	1
Early CABG*	68 (5.2%)	0 (0.0%)	0 (0.0%)	49 (10.8%)	19 (14.7%)	< 0.001	1	< 0.001	< 0.001	< 0.001	< 0.001	1
Death	62 (4.7%)	16 (2.6%)	11 (9.0%)	24 (5.3%)	11 (8.5%)	< 0.001	0.012	< 0.001	< 0.001	1	1	0.666
MI	48 (3.7%)	9 (1.5%)	5 (4.1%)	26 (5.7%)	8 (6.2%)	< 0.001	0.546	0.024	< 0.001	1	1	1
UAP	24 (1.8%)	4 (0.7%)	0 (0.0%)	16 (3.5%)	4 (3.1%)	< 0.001	1	< 0.001	0.480	0.204	1	1
Death, MI or UAP	134 (10.2%)	29 (4.8%)	16 (13.1%)	66 (14.5%)	23 (17.8%)	< 0.001	0.144	< 0.001	< 0.001	0.900	0.126	0.984
Annual adverse event rate (%) with 95% CI	1.8 (1.5–2.1)	0.8 (0.6–1.1)	2.3 (1.4–3.8)	2.6 (2.1–3.3)	3.2 (2.1–4.8)	< 0.001	< 0.001	< 0.001	< 0.001	0.638	0.308	0.408

BMI body mass index, ACEi angiotensin converting enzyme inhibitor, ARB angiotensin II receptor blockers, sMBF stress myocardial blood flow, PAV percent atheroma volume, NCPV percent non-calcified plaque volume, CPV percent calcified plaque volume, PCI percutaneous coronary intervention, CABG coronary artery bypass graft, MI myocardial infarction, UAP unstable angina pectoris. * Within 6 months after the CTA/PET imaging. (Categorical variables are presented as counts and percentages, continuous variables as median with interquartile ranges, except for annual adverse event rate (%) for which 95% confidence intervals are reported.)

Patients with diabetes were older, more frequently male, had higher body mass index (BMI), more often hypertension and dyslipidemia, and less often family history of premature CAD than non-diabetic patients, whereas typical angina pectoris was equally prevalent among diabetic and non-diabetic patients (Supplementary Table 1). Patients with abnormal perfusion had more often typical angina pectoris, were more frequently male, had more often diabetes and dyslipidemia and higher BMI as compared with patients having normal perfusion (Supplementary Table 1). Performance of early revascularization was highly associated with the presence of abnormal PET perfusion (Table 1 and Supplementary Table 1). Baseline medication is presented in Table 1 and Supplementary Table 1.

### Adverse clinical outcomes

**Fig. 2:**
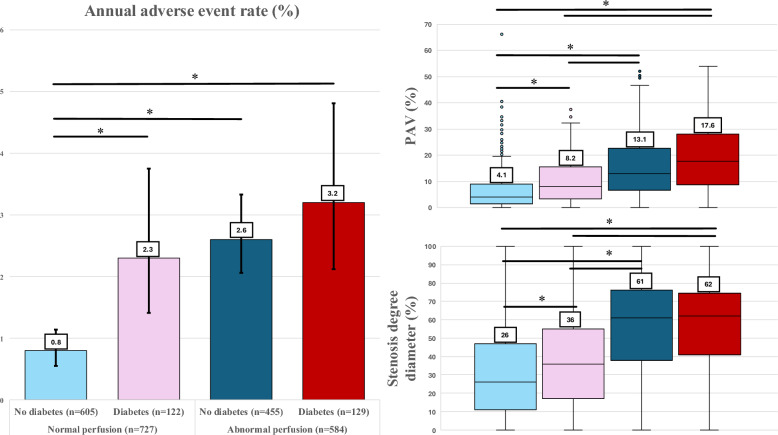
Bar chart (left) showing annual composite adverse event % rates (with 95% CI) stratified by myocardial perfusion and diabetes. Box plots (right) showing quantitative percent atheroma volume (PAV) and coronary diameter stenosis degree (median, 25-75th percentiles, and outliers similarly stratified by myocardial perfusion and diabetes. Comprehensive statistics are provided in Table 1. *Indicates statistical significance *p* < 0.05

During the long-term follow-up (median 7.0 years), 134 adverse events were recorded, including 62 deaths, 48 MIs and 24 UAPs. Detailed numbers of events are shown in Table 1 and Supplementary Table 1. The annual composite event rate was generally higher in diabetic than in non-diabetic patients (2.8% vs. 1.5%, *p* = 0.002). Likewise, event rate was higher in the presence of abnormal vs. normal myocardial perfusion (2.8% vs. 1.0%, *p* < 0.001). Patients with normal myocardial perfusion and without diabetes had the most favorable long-term outcome, with an annual composite event rate of 0.8%. Compared with them, patients with either diabetes (2.3%) or abnormal perfusion (2.6%) or both diabetes and abnormal perfusion (3.2%) had significantly higher annual adverse event rates despite having higher rate of revascularization performed (Fig. 2). The latter three groups showed no significant pair-wise differences in adverse event rate (Table 1).

### Quantified severity of CAD

Patients with diabetes had generally higher coronary artery plaque burden (PAV 11.8% vs. 7.1%, *p* < 0.001), higher degree of coronary stenosis diameter (52% vs. 38%, *p* < 0.001), more often obstructive CAD (55% vs. 43%, *p* < 0.001), and more often abnormal myocardial perfusion (51% vs. 43%, *p* = 0.015), as compared with non-diabetic patients (Supplementary Table 1). Among patients with normal myocardial perfusion, those with diabetes had two-fold total coronary plaque burden by PAV (8.2% vs. 4.1%, *p* < 0.001) as well as higher burden of non-calcified and calcified plaque components, and higher degree of coronary stenosis diameter (36% vs. 26%, *p* = 0.017), as compared with non-diabetic patients. Regional and global stress MBF were comparable between non-diabetic and diabetic patients within this normal perfusion subgroup (Table 1).

Compared with patients having normal myocardial perfusion, patients with abnormal perfusion had higher coronary plaque burden (PAV 13.8% vs. 4.6%, *p* < 0.001), higher degree of stenosis (62% vs. 27%, *p* < 0.001), more frequently obstructive CAD (68% vs. 26%, *p* < 0.001), and lower global and regional stress MBF (Supplementary Table 1).

Among patients having abnormal myocardial perfusion, patients with diabetes had similar plaque burden by PAV (17.6% vs. 13.1%, *p* = 0.121) and degree of coronary stenosis diameter (62% vs. 61%, *p* = 1.00) as non-diabetic patients. Regional sMBF and global sMBF were also comparable (Table 1).

Of note, non-diabetic patients with abnormal myocardial perfusion had significantly higher total PAV (13.1% vs. 8.2%, *p* < 0.001), NCPV (7.9% vs 5.4%, *p* < 0.001), and stenosis degree (61% vs. 36%, *p* < 0.001) in comparison with diabetic patients having normal perfusion, whereas CPV was comparable (3.9% vs. 2.8%, *p* = 0.178) **(**Table 1**)**.

### Predictors of adverse events

In Cox regression analysis, univariable predictors of composite outcome were increasing age, male sex, diabetes, quantitative CTA and PET imaging findings, and early revascularization **(**Table 2**)**. In addition to significant clinical predictors, only one PET parameter and one CTA parameter at a time were included in multivariable models, to avoid multicollinearity. In a multivariable Cox regression models in the total cohort (n = 1311), increasing total plaque burden by PAV, decreasing regional stress MBF (continuous variable) or presence of abnormal perfusion (binary variable), and increasing age were independent predictors of adverse outcome, whereas diabetes, sex, global stress MBF and early revascularization were not (Table 2). Interaction terms between diabetes and regional sMBF (*p* = 0.199) and between diabetes and PAV (*p* = 0.750) were non-significant when added to the adjusted multivariable Cox models (Supplemental Table 2). Testing of the proportional-hazards assumption revealed time-varying effects for age, sex, diabetes, and regional stress MBF (*p* < 0.05), resolved by stratified analyses allowing different baseline hazards for different groups (Table 3). Center-specific analyses were directionally consistent across centers although limited by statistical power (Supplementary Table 3).

**Table 2:** Univariable and multivariable Cox regression for composite adverse endpoint for the whole cohort (n = 1311)

Univariable predictors of the composite endpoint (MI/UAP/death) 7 years				Multivariable model with abnormal perfusion (binary)		Multivariable model with continuous regional sMBF		Multivariable model with continuous global sMBF	
Model	HR (95% CI)	*p*-value	*p*-value for interaction with diabetes	HR (95% CI)	*p*-value	HR (95% CI)	*p*-value	HR (95% CI)	*p*-value
Age (1-year increase)	1.05 (1.03–1.07	< 0.001	0.381	1.04 (1.02–1.07)	< 0.001	1.04 (1.02–1.07)	< 0.001	1.04 (1.02–1.07)	< 0.001
Male sex	1.75 (1.22–2.50)	0.002	0.742	1.35 (0.92–1.97)	0.126	1.31 (0.89–1.92)	0.176	1.35 (0.91–2.01)	0.132
Diabetes	1.79 (1.23–2.60)	0.002	N/A	1.38 (0.95–2.02)	0.092	1.39 (0.95–2.02)	0.090	1.38 (0.94–2.01)	0.097
BMI (1 kg/m^2^ increase)	1.00 (0.96–1.03)	0.819	0.178						
Hypertension	1.32 (0.93–1.88)	0.125	0.988						
Dyslipidemia	1.21 (0.86–1.72)	0.28 0	0.127						
Family history	0.81 (0.58–1.14)	0.235	0.542						
Current smoker	1.06 (0.71–1.58)	0.793	0.781						
Typical angina pectoris	1.12 (0.78–1.61)	0.546	0.859						
Abnormal perfusion (binary)	2.65 (1.85–3.79)	< 0.001	0.030	1.80 (1.19–2.73)	0.005				
Regional sMBF (0.1 ml/g/min decrease)	1.05 (1.03–1.07)	< 0.001	0.109			1.03 (1.01–1.06)	0.014		
Global sMBF (0.1 ml/g/min decrease)	1.04 (1.02–1.06)	< 0.001	0.006					1.02 (1.00–1.04)	0.132
Obstructive (≥ 50%) stenosis	3.05 (2.12–4.40)	< 0.001	0.271						
Stenosis degree diameter (1% increase)	1.02 (1.01–1.03)	< 0.001	0.122						
PAV (1% increase)	1.05 (1.04–1.06)	< 0.001	0.214	1.03 (1.02–1.05)	< 0.001	1.03 (1.02–1.05)	< 0.001	1.04 (1.02–1.05)	< 0.001
Early revascularization	1.76 (1.21–2.55)	0.003	0.033	0.86 (0.57–1.30)	0.476	0.81 (0.52–1.26)	0.350	0.91 (0.59–1.41)	0.677

BMI body mass index, sMBF stress myocardial blood flow, PAV percent atheroma volume

**Table 3:** Multivariable Cox regression for composite adverse outcome in subgroups

Multivariable Cox regression models				
	No diabetes		Diabetes	
Model	HR (95% CI)	*p*-value	HR (95% CI)	*p*-value
Age (1-year increase)	1.05 (1.02–1.07)	< 0.001	1.04 (1.00–1.08)	0.075
Male sex	1.13 (0.72–1.78)	0.602	1.87 (0.88–3.99)	0.104
PAV (1% increase)	1.03 (1.01–1.05)	< 0.001	1.04 (1.01–1.07)	0.014
Regional sMBF (0.1 ml/g/min decrease)	1.04 (1.01–1.07)	0.016	1.01 (0.97–1.06)	0.610
Early revascularization	1.03 (0.61–1.75)	0.908	0.47 (0.20–1.11)	0.086
	Normal perfusion		Abnormal perfusion	
Model	HR (95% CI)	*p*-value	HR (95% CI)	*p*-value
Age (1-year increase)	1.09 (1.05–1.13)	< 0.001	1.03 (1.00–1.05)	0.053
Male sex	1.61 (0.87–2.96)	0.129	1.14 (0.70–1.83)	0.604
Diabetes	2.39 (1.29–4.44)	0.006	1.09 (0.67–1.76)	0.729
PAV (1% increase)	1.06 (1.03–1.08)	< 0.001	1.02 (1.00–1.04)	0.021
Early revascularization	0.57 (0.13–2.40)	0.440	0.94 (0.61–1.46)	0.784

PAV percent atheroma volume, sMBF stress myocardial blood flow

Event-specific Cox regression analyses revealed that diabetes was an independent predictor of all-cause mortality whereas decreasing perfusion was an independent predictor of acute coronary syndromes (MI/UAP); PAV was an independent predictor of both these outcomes (Supplementary Table 3). In a 6-month landmark analysis PAV remained an independent predictor of the composite endpoint whereas the association of regional sMBF and long-term outcome was attenuated (Supplementary Table 3).

There was a statistically significant interaction between the presence of diabetes and abnormal perfusion (*p* = 0.030) in predicting adverse events. Therefore, multivariable Cox regressions were carried out separately in subgroups of patients with or without diabetes (Table 3). In patients without diabetes, both PAV and regional sMBF along with age were independent predictors of adverse events. In diabetic patients only PAV was a significant predictor whereas regional sMBF and age were not. Similarly, multivariable Cox regressions were performed in subgroups of patients with normal or abnormal perfusion (Table 3). In normal perfusion subgroup, age, diabetes and plaque burden by PAV were independent predictors of adverse events. In contrast, only plaque burden by PAV remained an independent predictor of events in patients with abnormal perfusion whereas diabetes was not (Table 3). Additional inclusion of regional sMBF as a continuous variable in the models did not affect the results.

**Fig. 3:**
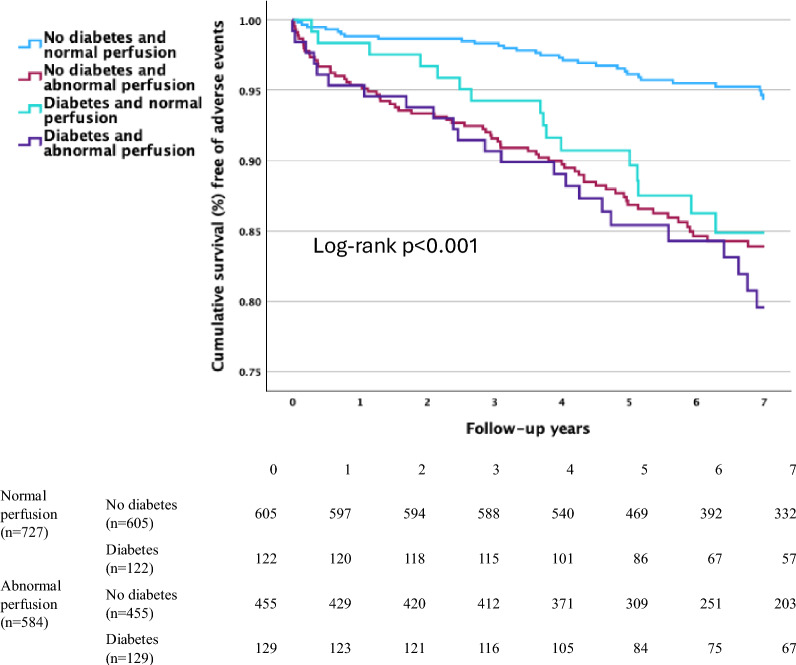
Kaplan–Meier survival curves for non-diabetic and diabetic patients stratified by binary PET perfusion finding

Kaplan–Meier curves (Fig. 3) show that event-free survival was significantly better in non-diabetic patients with normal stress perfusion as compared with the other groups (pairwise comparisons *p* < 0.001), whereas there were not significant differences between groups with either diabetes or abnormal stress perfusion or both.

## Discussion

In this study we describe a real-world patient cohort undergoing quantitative measurement of coronary atherosclerosis and myocardial perfusion, and assessment of clinical outcomes throughout long-term follow-up. The main findings can be summarized as follows: Non-diabetic patients with normal myocardial perfusion had favourable long-term outcomes whereas the presence of either diabetes or abnormal myocardial perfusion or both significantly increased the risk of adverse events, including death, MI, or UAP. Among patients with preserved myocardial perfusion, those with diabetes had a twofold coronary atherosclerotic plaque burden as compared with non-diabetic patients, potentially explaining their worse long-term clinical outcome. In contrast, patients with abnormal myocardial perfusion had similarly increased long-term risk of adverse events irrespective of the presence of diabetes. Coronary atherosclerotic plaque burden appeared as a significant predictor of long-term outcome in all subgroup analyses.

Combination of anatomical and functional imaging modalities, such as coronary CTA and PET MPI, enables comprehensive phenotyping of CAD with CTA visualizing atherosclerotic plaques and PET assessing their functional consequences on blood flow. Previously we have shown, in a cohort partly overlapping with the current study, that these non-invasive imaging findings are useful for risk stratification both in non-diabetic and diabetic patients with suspected CAD [35]. For the current observational cohort study including patients from two centres, coronary CTA scans were re-analyzed in a blinded manner to measure coronary atherosclerotic plaque burden quantitatively as PAV. Likewise, stress MBF was quantified from PET MPI. Hence, the current study is unique in allowing us to study both the anatomical and functional quantitative details of CAD in relation to diabetes.

In a previous large registry study by Olesen et al., patients with diabetes had 1.3–1.6-fold crude rates for MI, cardiac death, and all-cause death as compared with non-diabetic patients in the absence of angiographically obstructive coronary artery stenosis, but adjustment by additional variables rendered most of these differences non-significant [7]. However, longer follow-up (median 8.8 years) in the mainly overlapping cohort showed that diabetes was associated with increased all-cause mortality rates even in the absence of angiographical CAD [36]. Among patients with normal myocardial perfusion, we found a significantly higher annual rate of the composite endpoint in diabetic vs. non-diabetic patients (2.3% vs. 0.8%), and the presence of diabetes remained a significant predictor of outcome after multivariable adjustment (HR 2.4). In addition, PAV was a significant independent predictor of outcome in this patient subgroup with normal perfusion where PAV was approximately twofold in diabetic vs. non-diabetic patients. These findings suggest that the increased event rate in diabetic patients with preserved myocardial perfusion is partly explained by more advanced but non-obstructive coronary atherosclerosis, and may indicate the importance of anatomical assessment by CTA in risk stratification of diabetic patients even if myocardial perfusion appears normal. However, comparing our results with those by Olesen et al., it is important to note that normal myocardial perfusion does not equal to the absence of obstructive coronary stenosis, especially as our study cohort was partly selected based on obstructive coronary CTA findings possibly rendering the event rates generally higher.

Murthy et al. reported a very low (0.3%) annual rate of cardiac mortality in diabetic patients with preserved myocardial flow reserve by ^82^Rb PET and without known CAD [17]. This is different from our study where PET perfusion imaging was partly triggered by visual obstructive coronary CTA findings and a composite endpoint of all-cause mortality, MI, and UAP was recorded, potentially explaining the higher event rates observed in our study. Of note, we used regional assessment of stress MBF whereas Murthy et al. measured global flow reserve. Assante et al. used ^82^Rb PET perfusion imaging and propensity score matching in their study and reported that the presence of either diabetes or reduced myocardial flow reserve was associated with threefold event rates as compared with the absence of both, consistent with our findings [37]. However, the annual event rates in absolute terms were remarkably lower than in our study that could be related to the exclusion of patients with obstructive CAD or visual perfusion defects in their study. In turn, Caobelli et al. studied asymptomatic diabetic patients using single photon emission computed tomography (SPECT) MPI and found a low event rate with a normal semi-quantitative SPECT finding whereas the presence of a perfusion defect was associated with a threefold event rate during 5-year follow-up [38].

The relationship between diabetes and CAD detected by coronary CTA was previously studied in the observational CONFIRM registry where 1823 diabetic patients were propensity-matched with non-diabetic patients. The investigators found that diabetic patients had about twofold all-cause mortality rate during the 5-year follow-up as compared with non-diabetic patients in the setting of non-obstructive CAD or obstructive CAD on coronary CTA, whereas no significant outcome difference according to diabetes status was observed in the absence of coronary atherosclerosis on CTA [9]. Interestingly, diabetic patients with non-obstructive CAD had outcomes comparable to non-diabetic patients with multi-vessel obstructive CAD. In a large Danish registry study patients were similarly stratified as having no CAD, non-obstructive CAD, or obstructive CAD based on coronary CTA [8]. Patients with diabetes were shown to have significantly higher all-cause mortality rates than non-diabetic patients, irrespective of the CAD severity. In contrast, the rate of MI during the 3.6-year median follow-up was not significantly different between diabetic and non-diabetic patients who had no CAD or non-obstructive CAD.

A recent analysis from the SCOT-HEART trial included 1769 patients who underwent quantitative CTA analysis of coronary atherosclerotic plaques [39]. Diabetic patients as compared with non-diabetic had higher coronary artery calcium score and higher quantitated total plaque volume and different plaque components, whereas there was no difference in the prevalence of obstructive coronary stenosis or visually assessed adverse plaque characteristics. A report by Jonas et al. from the CREDENCE trial included 303 patients whose coronary CTA images were quantitatively analyzed by AI-QCT algorithm, i.e., the same software tool that was used in our study [40]. They found that among patients with non-obstructive CAD, those with diabetes had higher quantitated plaque volumes than those without diabetes. Moreover, total plaque volume and different plaque components were comparable between non-diabetic patients with obstructive stenosis and diabetic patients with non-obstructive stenosis. In contrast, we found higher total and non-calcified plaque volumes and higher stenosis degree in non-diabetic patients with abnormal myocardial perfusion as compared with diabetic patients who had normal perfusion. This reflects the fact that patients with reduced myocardial perfusion have advanced coronary atherosclerosis, whereas not all angiographically obstructive stenoses are ischemia-inducing. Our study extends the previous evidence by providing long-term patient follow-up and showing that coronary atherosclerotic plaque burden measured by PAV is an important prognostic marker: both in subgroups of normal and abnormal myocardial perfusion and in subgroups of non-diabetic and diabetic patients. This is biologically plausible as the increasing amount of atherosclerotic plaque means a larger pool of potentially vulnerable plaques and therefore a substrate for long-term adverse events.

Recently, the extent of coronary atherosclerosis measured as the number of segments with plaque on CTA (segment involvement score) was found to provide incremental prognostic information over stenosis severity and presence of perfusion defect in diabetic patients undergoing hybrid coronary CTA and single photon emission computerized tomography MPI [15]. The extent of CAD in our study is represented by plaque burden by PAV. Interestingly, in our multivariable models neither regional nor global perfusion findings were independent predictors of events in diabetic patients, being in line with our observation of impaired outcome in diabetic patients despite preserved myocardial perfusion. This may be partly related to long follow-up time (7 years) in our study since our landmark analysis suggested that perfusion abnormalities mainly inform about short-term risk whereas anatomical plaque burden retains prognostic value over long-term follow-up.

### Limitations

Although the cohort was reasonably sized and the follow-up time was up to 7 years, the number of adverse events remained moderately low, limiting the statistical power in subgroup analyses. For the same reason we assessed composite adverse endpoint including mortality, MI, and UAP, rather than each event type separately. Noteworthily, the clinical events were manually confirmed by the members of the study group, however, without formal blinding. Furthermore, this study was retrospective, and some information were not available, most importantly the presence of diabetic kidney disease, duration of diabetes, glycemic control, insulin resistance, and drug classes, precluding adjustment for these factors. In some cases the presence of diabetes was uncertain. Thus, patients with unknown diabetes status were excluded. Causes of death were not available, and therefore, we assessed all-cause mortality that is free from verification bias. Early revascularization was predominantly planned based on CTA/PET imaging findings and could alter the outcomes; therefore, early revascularization was included in multivariable models, and its confounding effect was further addressed in a landmark analysis. In our study we focused on total plaque burden as a comprehensive measure of coronary atherosclerosis, integrating both calcified and non-calcified components and known to contribute to long-term risk [41].

Importantly, selective hybrid imaging protocol was used in the Turku University Hospital where a suspected obstructive (≥ 50%) coronary stenosis on CTA triggers downstream PET perfusion imaging. This approach is supported by the current guidelines which recommend coronary CTA as a first-line diagnostic test option in patients with low or moderate clinical likelihood of CAD. Based on guidelines this can be followed by the selective use of functional imaging for evaluating the hemodynamic significance of stenosis [11]. However, this approach can introduce selection bias due to higher prevalence of atherosclerosis, although affecting both non-diabetic and diabetic patients. However, the study cohort was merged from two centers, which potentially improves the generalizability of the findings: in the final study cohort less than half (45%) of the patients had obstructive CAD based on AI-QCT, and therefore, we think our cohort reasonably represents the continuum of CAD. Most patients underwent stress-only MPI protocol, and therefore, the absence of rest MBF, myocardial flow reserve, or dedicated microvascular indices is a limitation of our study. Finally, patients were symptomatic, and results cannot be generalized into asymptomatic patients.

## Conclusions

Diabetes is associated with anatomically and functionally more advanced CAD. Myocardial perfusion risk-stratifies non-diabetic patients, whereas diabetic patients have impaired long-term outcome irrespective of perfusion findings, partly attributable to higher coronary plaque burden. Quantified burden of coronary atherosclerosis measured by PAV predicts long-term outcome both in non-diabetic and diabetic patients.

## Supplementary Information

Supplementary Material 1

## Data Availability

The data that support the findings of this study are available on reasonable request from the corresponding author. The data are not publicly available due to privacy and ethical restrictions.

## Abbreviations

AI-QCT: Artificial intelligence-guided quantitative computed tomography
CPV: Calcified plaque volume
CTA: Computed tomography angiography
CABG: Coronary artery bypass graft
CAD: Coronary artery disease
ICA: Invasive coronary angiography
MBF: Myocardial blood flow
MI: Myocardial infarction
MPI: Myocardial perfusion imaging
NCPV: Non-calcified plaque volume
PAV: Percent atheroma volume
PCI: Percutaneous coronary intervention
PET: Positron emission tomography
SPECT: Single photon emission computed tomography
sMBF: Stress myocardial blood flow
UAP: Unstable angina pectoris
