# Supplementary material for: Prognostic implications of quantified coronary atherosclerosis and myocardial perfusion in diabetes
Source: Cardiovasc Diabetol. 2025 Dec 2;24:453. doi: 10.1186/s12933-025-03006-x (PMC12676773; doi:10.1186/s12933-025-03006-x)
Supplement: Supplementary file 1 — Supplementary Material 1 [file 12933_2025_3006_MOESM1_ESM.docx]

***Supplementary table 1. Patient characteristics stratified by diabetes and myocardial perfusion separately.***

| **Patient characteristics** | | | | | | | |
| --- | --- | --- | --- | --- | --- | --- | --- |
|  | Total cohort | No diabetes | Diabetes | Overall p-value | Normal perfusion | Abnormal perfusion | Overall p-value |
| N | 1311 (100%) | 1060 (80.9%) | 251 (19.1%) |  | 727 (55.5%) | 584 (44.5%) |  |
| **Baseline characteristics** | | | | | | | |
| Age (years) | 63 (56-69) | 62 (56-68) | 64 (58-69) | 0.045 | 63 (56-69) | 63 (56-69) | 0.590 |
| BMI (kg/m^2^) | 27.1 (24.6-30.1) | 26.4 (24.2-29.3) | 29.8 (27.1-34.0) | <0.001 | 26.8 (24.2-29.8) | 27.5 (24.8-30.4) | 0.018 |
| Male sex | 713 (54.4%) | 559 (52.7%) | 154 (61.4%) | 0.014 | 297 (40.9%) | 416 (71.2%) | <0.001 |
| Diabetes | 251 (19.1%) |  |  |  | 122 (16.8%) | 129 (22.1%) | <0.001 |
| Hypertension | 767 (58.5%) | 576 (54.3%) | 191 (76.1%) | <0.001 | 419 (57.6%) | 348 (59.6%) | 0.475 |
| Dyslipidemia | 757 (57.7%) | 578 (54.5%) | 178 (70.9%) | <0.001 | 399 (54.9%) | 357 (61.1%) | 0.023 |
| Family history | 664 (50.6%) | 569 (53.7%) | 95 (37.8%) | <0.001 | 388 (53.4%) | 276 (47.3%) | 0.028 |
| Current smoker | 285 (21.7%) | 233 (22.0%) | 52 (20.7%) | 0.662 | 144 (19.8%) | 141 (24.1%) | 0.058 |
| Typical angina pectoris | 392 (29.9%) | 321 (30.3%) | 71 (28.3%) | 0.535 | 185 (25.4%) | 207 (35.4%) | <0.001 |
| **Baseline medications** | | | | | | | |
| Beta-blocker | 745 (56.8%) | 585 (55.2%) | 160 (63.7%) | 0.014 | 390 (53.6%) | 355 (60.8%) | 0.009 |
| Lipid-lowering drug | 755 (57.6%) | 566 (53.4%) | 189 (75.3%) | <0.001 | 386 (53.1%) | 369 (63.2%) | <0.001 |
| Antiplatelet drug | 817 (62.3%) | 656 (61.9%) | 161 (64.1%) | 0.507 | 424 (58.3%) | 393 (67.3%) | <0.001 |
| Long-acting nitrate | 81 (6.2%) | 52 (4.9%) | 29 (11.6%) | <0.001 | 44 (6.1%) | 37 (6.3%) | 0.832 |
| ACEi or ARB | 538 (41.0%) | 378 (35.7%) | 160 (63.7%) | <0.001 | 273 (37.6%) | 265 (45.4%) | 0.004 |
| Calcium channel blocker | 301 (23.0%) | 219 (20.7%) | 82 (32.7%) | <0.001 | 152 (20.9%) | 149 (25.5%) | 0.049 |
| **Imaging findings** | | | | | | | |
| Abnormal perfusion | 584 (44.5%) | 455 (42.9%) | 129 (51.4%) | 0.015 |  | | |
| Regional sMBF (ml/g/min) | 2.32 (1.58-2.95) | 2.36 (1.60-3.02) | 2.13 (1.46-2.68) | 0.001 | 2.87 (2.52-3.38) | 1.51 (1.07-1.87) | <0.001 |
| Global sMBF (ml/g/min) | 3.00 (2.22-3.75) | 3.05 (2.28-3.83) | 2.78 (1.98-3.55) | <0.001 | 3.64 (3.19-4.23) | 2.10 (1.70-2.55) | <0.001 |
| Stenosis degree diameter (%) | 42 (18-64) | 38 (17-64) | 52 (23-70) | <0.001 | 27 (12-50) | 62 (38-75) | <0.001 |
| Obstructive (≥50%) stenosis | 590 (45.0%) | 452 (42.6%) | 138 (55.0%) | <0.001 | 192 (26.4%) | 398 (68.2%) | <0.001 |
| PAV (%) | 7.8 (2.6-16.5) | 7.1 (2.1-15.0) | 11.8 (5.2-22.3) | <0.001 | 4.6 (1.5-9.8) | 13.8 (7.0-23.6) | <0.001 |
| NCPV (%) | 5.1 (2.1-9.1) | 4.6 (1.7-8.5) | 7.6 (3.5-11.4) | <0.001 | 3.1 (1.3-5.9) | 8.3 (4.9-12.6) | <0.001 |
| CPV (%) | 2.0 (0.2-6.2) | 1.7 (0.2-5.6) | 3.7 (0.8-8.9) | <0.001 | 1.0 (0.0-3.5) | 4.2 (1.0-10.1) | <0.001 |
| **Follow-up (up to 7 years)** | | | | | | | |
| Early PCI OR CABG* | 269 (20.5) | 203 (19.2%) | 66 (26.3%) | 0.012 | 29 (4.0%) | 240 (41.1%) | <0.001 |
| Early PCI* | 201 (15.3%) | 154 (14.5%) | 47 (18.7%) | 0.097 | 29 (4.0%) | 172 (29.5%) | <0.001 |
| Early CABG* | 68 (5.2%) | 49 (4.6%) | 19 (7.6%) | 0.058 | 0 (0.0%) | 68 (11.6%) | <0.001 |
| Death | 62 (4.7%) | 40 (3.8%) | 22 (8.8%) | 0.003 | 27 (3.3%) | 35 (6.0%) | <0.001 |
| MI | 48 (3.7%) | 35 (3.3%) | 13 (5.2%) | 0.216 | 14 (1.9%) | 34 (5.8%) | 0.002 |
| UAP | 24 (1.8%) | 20 (1.9%) | 4 (1.6%) | 0.960 | 4 (0.6%) | 20 (3.4%) | <0.001 |
| Death, MI or UAP | 134 (10.2%) | 95 (9.0%) | 39 (15.5%) | 0.002 | 45 (6.2%) | 89 (15.2%) | <0.001 |
| Annual adverse event rate (%) with 95% CI | 1.8% (1.5-2.1) | 1.5% (1.3-1.9) | 2.8% (2.0-3.8) | 0.002 | 1.0% (0.8-1.4) | 2.8% (2.2-3.4) | <0.001 |

*BMI body mass index, ACEi angiotensin converting enzyme inhibitor, ARB angiotensin II receptor blockers, sMBF stress myocardial blood flow, PAV percent atheroma volume, NCPV percent non-calcified plaque volume, CPV percent calcified plaque volume, PCI percutaneous coronary intervention, CABG coronary artery bypass graft, MI myocardial infarction, UAP unstable angina pectoris. * Within 6 months after the CTA/PET imaging. (Categorical variables are presented as counts and percentages, continuous variables as median with interquartile ranges, except for annual adverse event rate (%) for which 95% confidence intervals are reported.)*

***Supplementary table 2. Multivariable Cox regression analysis for adjusted interaction terms for diabetes and quantitative imaging parameters***

| Multivariable model with continuous regional sMBF (adjusted interaction) | | |
| --- | --- | --- |
| Model ​ | HR (95% CI) ​ | p-value ​ |
| Age (1-year increase) ​ | 1.04 (1.02-1.07) | <0.001 |
| Male sex ​ | 1.29 (0.88-1.07) | 0.193 |
| Early revascularization | 0.82 (0.53-1.28) | 0.375 |
| Diabetes​ | 0.86 (0.23-3.25) | 0.828 |
| Regional sMBF (0.1 ml/g/min decrease) | 1.04 (1.01-1.07) | 0.007 |
| Diabetes*Regional sMBF (0.1 ml/g/min decrease) | 0.97 (0.93-1.02) | 0.199 |
| PAV (1% increase) | 1.04 (1.02-1.05) | <0.001 |
| Diabetes*PAV (1% increase) | 1.00 (0.96-1.03) | 0.750 |

*sMBF stress myocardial blood flow, PAV percent atheroma volume*

***Supplementary Table 3.*** Separate multivariable Cox regression analyses for different centers (Turku and Amsterdam), different clinical endpoints (all-cause mortality and MI/UAP), and a landmark analysis excluding patients who experienced an event withing 6 months from CTA/PET imaging.

|  | Multivariable model with continuous regional sMBF (endpoint death) | | Multivariable model with continuous regional sMBF (endpoint MI/UAP) | | Multivariable model with continuous regional sMBF (subcohort Turku) | | Multivariable model with continuous regional sMBF (subcohort Amsterdam) | | Multivariable model with continuous regional sMBF (0.5-7 years follow-up) | |
| --- | --- | --- | --- | --- | --- | --- | --- | --- | --- | --- |
| Model ​ | HR (95% CI) ​ | p-value ​ | HR (95% CI) ​ | p-value ​ | HR (95% CI) ​ | p-value ​ | HR (95% CI) ​ | p-value ​ | HR (95% CI) ​ | p-value ​ |
| Age (1-year increase) ​ | 1.07 (1.04-1.11) | <0.001 | 1.01 (0.98-1.06) | 0.383 | 1.04 (1.01-1.07) | 0.007 | 1.05 (1.00-1.08) | 0.013 | 1.06 (1.03-1.08) | <0.001 |
| Male sex ​ | 1.51 (0.89-2.57) | 0.124 | 0.99 (0.58-1.69) | 0.965 | 1.24 (0.79-1.94) | 0.343 | 1.63 (0.75-3.57) | 0.218 | 1.32 (0.86-2.03) | 0.199 |
| Diabetes​ | 1.82 (1.11-3.00) | 0.018 | 1.02 (0.59-1.79) | 0.933 | 1.34 (0.84-2.12) | 0.216 | 1.57 (0.80-3.06) | 0.187 | 1.45 (0.95-2.20) | 0.083 |
| Regional sMBF (0.1 ml/g/min decrease) | 1.03 (0.99-1.06) | 0.139 | 1.04 (1.01-1.08) | 0.020 | 1.03 (1.00-1.06) | 0.029 | 1.03 (0.98-1.08) | 0.247 | 1.03 (1.00-1.05) | 0.067 |
| PAV (1% increase​) | 1.03 (1.00-1.05) | 0.025 | 1.04 (1.02-1.06) | <0.001 | 1.04 (1.02-1.06) | <0.001 | 1.02 (0.99-1.05) | 0.237 | 1.03 (1.01-1.05) | <0.001 |
| Early revascularization | 0.64 (0.34-1.21) | 0.172 | 0.99 (0.55-1.80) | 0.972 | 0.73 /0.43-1.25) | 0.257 | 1.00 (0.44-2.28) | 0.996 | 0.79 (0.47-1.30) | 0.346 |

*sMBF stress myocardial blood flow, PAV percent atheroma volume*
